# Rigidity and Flexibility: The Central Basis of Inter-Leg Coordination in the Locust

**DOI:** 10.3389/fncir.2016.00112

**Published:** 2017-01-11

**Authors:** Daniel Knebel, Amir Ayali, Hans-Joachim Pflüger, Jan Rillich

**Affiliations:** ^1^Department of Zoology, Tel Aviv UniversityTel Aviv, Israel; ^2^Sagol School of Neuroscience, Tel Aviv UniversityTel Aviv, Israel; ^3^Institut für Biologie, Neurobiologie, Freie Universität BerlinBerlin, Germany

**Keywords:** locust, locomotion, motor control, central pattern generator, intersegmental coordination, cross-spectrum analysis

## Abstract

Many motor behaviors, and specifically locomotion, are the product of an intricate interplay between neuronal oscillators known as central pattern generators (CPGs), descending central commands, and sensory feedback loops. The relative contribution of each of these components to the final behavior determines the trade-off between fixed movements and those that are carefully adapted to the environment. Here we sought to decipher the endogenous, default, motor output of the CPG network controlling the locust legs, in the absence of any sensory or descending influences. We induced rhythmic activity in the leg CPGs in isolated nervous system preparations, using different application procedures of the muscarinic agonist pilocarpine. We found that the three thoracic ganglia, each controlling a pair of legs, have different inherent bilateral coupling. Furthermore, we found that the pharmacological activation of one ganglion is sufficient to induce activity in the other, untreated, ganglia. Each ganglion was thus capable to impart its own bilateral inherent pattern onto the other ganglia via a tight synchrony among the ipsilateral CPGs. By cutting a connective and severing the lateral-longitudinal connections, we were able to uncouple the oscillators’ activity. While the bilateral connections demonstrated a high modularity, the ipsilateral CPGs maintained a strict synchronized activity. These findings suggest that the central infrastructure behind locust walking features both rigid elements, which presumably support the generation of stereotypic orchestrated leg movements, and flexible elements, which might provide the central basis for adaptations to the environment and to higher motor commands.

## Introduction

A common feature of animal locomotion is that of the underlying infrastructure of oscillators known as central pattern generators (CPGs). CPGs are neuronal circuits that can produce rhythmic motor output even in the absence of sensory feedback (recent reviews by Marder and Bucher, 2007; Mulloney and Smarandache, 2010; Marder, 2012; Rybak et al., 2015). A major question regarding CPG networks relates to the extent of their relative contribution to the final, adequate, motor behavior. Since most locomotion patterns involve the orchestrated rhythmic movements of different body parts controlled by discrete CPGs, an evaluation of the functional connectivity and inter-CPG orchestration is crucial for understanding their role in locomotor behaviors. Here we explored the interplay of the leg CPGs, the strictly central aspect of locomotion, in the desert locust *Schistocerca gregaria.*

Evidence for the instrumental role of inter-CPG connections has been shown across species and locomotion types. Pioneering work on the crustacean swimmeret system has revealed that interneurons couple CPGs to preserve the posterior-to-anterior wave of power-strokes in a bilateral synchrony that propels the body forward in water (e.g., Mulloney, 2003; for review: Mulloney and Smarandache-Wellmann, 2012). In undulatory swimming models, including both invertebrates and vertebrates, interneurons that interconnect CPGs determine the temporal characteristics of the rhythmic output. Thus, in the lamprey, the tadpole, and the leech, interneurons maintain a phase-lagged activity between the CPGs in order to coordinate an anterior-to-posterior body contraction (e.g., lamprey: McClellan and Grillner, 1984; Ayali et al., 2007; tadpole: Kahn and Roberts, 1982; Li et al., 2010; leech: Friesen and Hocker, 2001). Due to these inter-CPG connections, the sensory-deprived nervous systems of these models are capable of generating fictive swimming when pharmacologically or electrically stimulated (for review: Skinner and Mulloney, 1998).

Whereas swimming takes place in a relatively homogenous medium, multi-legged terrestrial locomotion raises additional challenges as CPG networks need to orchestrate their activities in regard to environmental inconsistencies (for review: Büschges et al., 2011). In addition, inter-leg coordination frequently changes on-the-move as animals alter their walking gaits (Wosnitza et al., 2013). Therefore, a major requirement of walking behavior is to provide an efficient solution for the trade-off between the stereotypic leg movements that propel the body forward, and the flexibility required for adequate performance. An excellent model for studying this compromise is that of insect walking.

Insect walking behavior is remarkable for its combination of stability, adaptability, and speed (e.g., for locusts: Burns, 1973; Pearson and Franklin, 1984; Duch and Pflüger, 1995). The underlying motor control of insect walking provides an integration of sensory feedback loops and central components (locust: Runion and Usherwood, 1968; Usherwood et al., 1968; Usherwood and Runion, 1970; Bräunig and Hustert, 1985; Laurent and Burrows, 1988; Matheson and Field, 1995; Newland and Emptage, 1996; for review on stick insect and cockroach: Ayali et al., 2015a). However, the way by which an insect shapes and maintains its inter-leg coordination is not fully understood, specifically with regard to the contribution of the different control elements (central or sensory).

Research from different insect preparations has provided some insights into the inter-leg couplings. In locusts, a well-established model of pattern generation (e.g., Ayali and Lange, 2010), interneurons were found to disperse among the segments proprioceptive information derived from the legs (MacMillan and Kien, 1983; Laurent, 1986; Laurent and Burrows, 1988). Studies of the stick insect walking system revealed that a single stepping leg can induce in-phase activity in neighboring leg CPGs, suggesting that CPG-CPG connections, intersegmental feedback loops, or their combination, allow the recruitment of neighboring motor circuits (Büschges, 2005; Ludwar et al., 2005; Borgmann et al., 2007). Recent studies in the cockroach and the fly have shown that when deprived of walking-related proprioceptive feedback, these insects largely walk normally (cockroach: Couzin-Fuchs et al., 2015; Drosophila: Mendes et al., 2013), thereby suggesting that inter-leg coordination is not solely dependent on sensory feedback loops (Ayali et al., 2015a,b; see also David et al., 2016).

Several studies have analyzed the hardwired coupling among the leg CPGs in deafferented or isolated nervous systems using topical application of the muscarinic agonist pilocarpine to activate the oscillators (locust: Ryckebusch and Laurent, 1993; Rillich et al., 2013; stick insect: Büschges et al., 1995; Ludwar et al., 2005; cockroach: Fuchs et al., 2011, 2012; David et al., 2016). In stick insects, central coupling was at most weak (Büschges et al., 1995; Ludwar et al., 2005), while in locusts, some examples of functional CPG-CPG crosstalk were reported but the coupling was highly variable (Ryckebusch and Laurent, 1994). In the cockroach and the moth, pilocarpine applied to an isolated nervous system produced a fictive-tripod pattern (cockroach: Fuchs et al., 2011; David et al., 2016; moth: Johnston and Levine, 2002). However, sensory information was found to enhance the fictive pattern of the cockroach (Fuchs et al., 2011, 2012), while the moth barely uses the tripod gait when walking. The various, and at times controversial, reports call for a comprehensive study of the central connections that serve as infrastructure for insect walking.

In this study we sought to decipher the central functional connectivity of the thoracic leg CPGs in the locust. To this end, we chose a reductionist approach to study these oscillators in a sensory-deprived nervous system, isolated in a dish. This approach involves the challenge of bridging the gap between the *in vitro* motor output and the natural behavior. Nonetheless, it is the only approach that provides direct insights into the inter-CPG connectivity without the possible masking of sensory or modulatory inputs. The underlying assumption that directed our study was that features of the networks observed and deduced in our experiments are the product of interneuronal activity that would necessarily have a role in the final behavior. Our findings thus contribute to understanding the underlying infrastructure of insect walking, while also signifying the role of descending and sensory mechanisms in generating a behaviorally relevant and variable output from the default hardwired connectivity.

Carefully designed experiments allowed us to investigate the neuronal control elements of the legs (the three thoracic ganglia: pro-, meso-, and metathoracic ganglia, from rostral to caudal), and their interactions. We revealed that the different ganglia exhibit different endogenous bilateral coupling, and that while contralateral connections are adjustable, ipsilateral synchrony dominates the coupling pattern. Our data suggest that the inter-leg coordination scheme in the locust includes both rigid and modular parts, which provide the hardwired central basis for walking stability and flexibility.

## Materials and Methods

### Experimental Animals

All experiments were performed on adult male desert locusts (*Schistocerca gregaria*, Forskål) from our colony at Tel Aviv University (Ayali and Zilberstein, 2002), within the first 2 weeks after the final molt. All experiments complied with the Principles of Laboratory Animal Care and the Israeli Law regarding the protection of animals.

### Preparation

Motor patterns were recorded either from *in vitro* individual thoracic ganglia or from *in vitro* thoracic ganglia chain preparations, including the pro-, meso-, and metathoracic ganglia. The animals were anesthetized with CO_2_ for at least 5 min prior to dissection. Following decapitation and the removal of appendages, the pronotal shield, and the abdomen posteriorly to the fourth abdominal segment, a longitudinal cut in the cuticle was performed along the dorsal midline of the thorax. The preparation was attached to a Sylgard dish (Sylgard 182 silicon Elastomer, Dow Corning Corp., Midland, MI, USA), and the cut was widened and superfused with locust saline containing (in mM): 150 NaCl, 5 KCl, 5 CaCl_2_, 2 MgCI_2_, 10 Hepes, 25 sucrose at pH 7.4. Air sacs and fatty tissue covering the ventral nerve cord were removed, and the thoracic ganglia chain with its surrounding tracheal supply was dissected out of the body cavity, pinned in a clean Sylgard dish, dorsal side up, and bathed in locust saline. The two main tracheae were opened and floated on the saline surface. Unless stated otherwise, all peripheral nerve branches originating from the thoracic ganglia were cut short except for nerve 5A (numbered after Campbell, 1961) that contains three motor axons: the slow and fast trochanteral depressors and a common inhibitor.

### Electrophysiological Recording

We used custom-made suction electrodes to record extracellularly the activity of the 5A nerves, unless stated otherwise. To record the pro-mesothoracic connective, we used a hook electrode. Recordings started 5 min before and lasted for at least 40 min after pilocarpine bath-application. Data were acquired and stored on the computer for off-line analysis using two four-channel differential amplifiers (Model 1700, A-M Systems, USA) and Axon Digidata 1440A A-D board with Axo-Scope software (Molecular Devices, Sunnyvale, CA, USA).

### Pharmacological Treatments

The muscarinic receptor agonist pilocarpine hydrochloride (Sigma–Aldrich, Deisenhofen, Germany) was dissolved in locust saline to a final concentration of 0.5 mM, which typically elicits rhythmic motor activity in leg motor nerves (Ryckebusch and Laurent, 1993). In two of the experimental conditions pilocarpine was applied into the bath to act directly on single individual thoracic ganglia or on all thoracic ganglia in an interconnected chain. In experiments with restricted drug-application, a petroleum jelly (Vaseline) barrier was built around one of the three interconnected thoracic ganglia. After leak-proofing, saline was applied into the Vaseline well, and after 5 min it was gently replaced with the pilocarpine solution. All other thoracic ganglia were bathed in normal saline only.

### Data Analysis

Overall, 58 experiments were performed in this study, each employing between two and five electrodes, to finally obtain 217 recordings of depressor motor nerve rhythmic patterns. Since the rhythmic pattern induced by pilocarpine requires several minutes to become established, we routinely evaluated the motor activity of the last 8 min of each experiment. We identified bursts based on instantaneous spike frequency, and measured a mean of 62 ± 19 burst cycles (*n* = 217). To evaluate the characteristics of the rhythmic patterns, the following parameters were measured using DataView Software (University of St Andrews; UK): instantaneous cycle frequency, cycle period and burst duration of depressor motor units, and the duty cycle (burst duration/cycle period). We averaged these parameters for each experiment and compared between the different experimental conditions. To test normality, we used the Kolmogorov–Smirnov test before using ANOVA to compare the groups. In some cases, log10 transformation was needed to normalize the data. In cases in which the data could not be normalized we used the Kruskal–Wallis test. All tests were followed by *post hoc* tests. All statistical tests were performed in GraphPad Prism 5 (GraphPad software Inc., San Diego, CA, USA).

**FIGURE 1:**
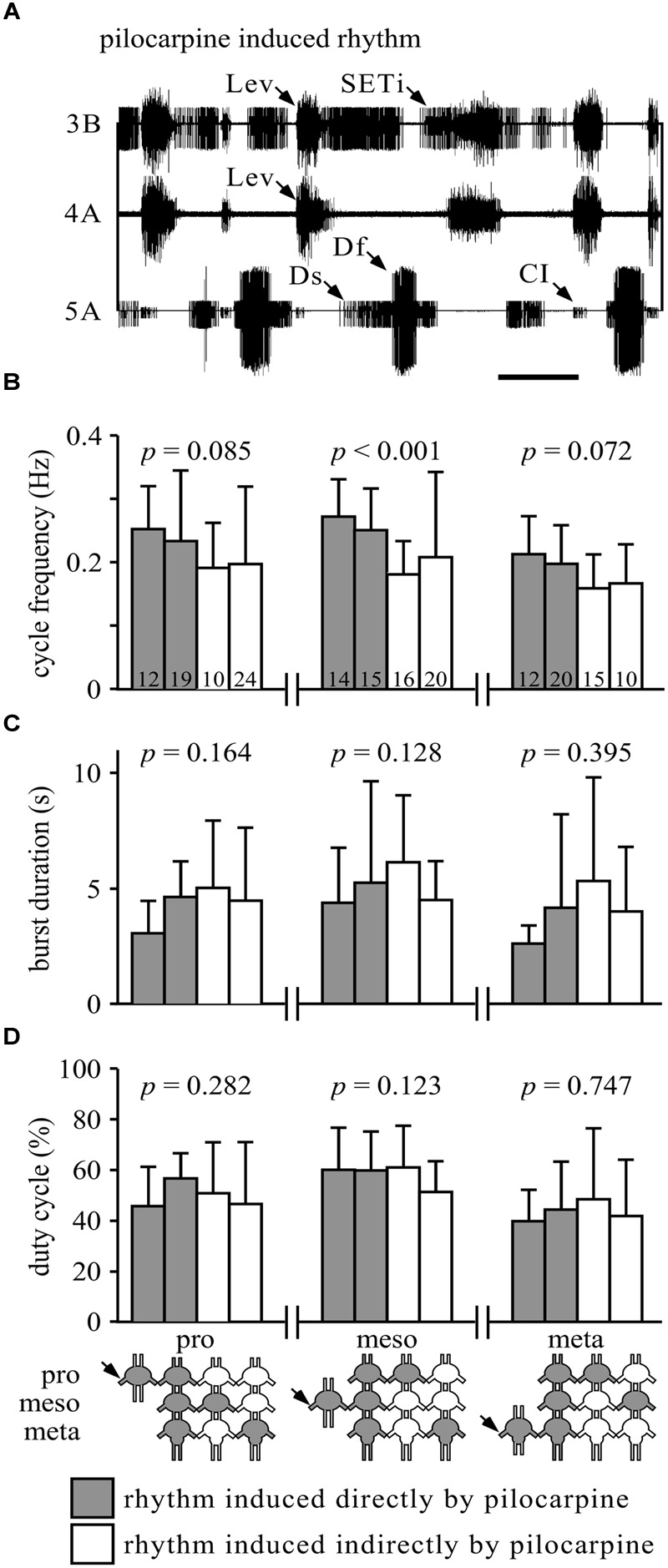
**Similarity of burst properties under different pharmacological stimulation methods. (A)** Simultaneous extracellular recordings of alternating burst activities in antagonistic trochanteral levator (nerve 3B and nerve 4A) and depressor MN (nerve 5A) of the metathoracic ganglion after Pilocarpine application. Lev, levators; SETi, slow extensor tibiae; Ds, slow depressor; Df, fast depressor; CI, Common Inhibitor. Scale bar: 1 s. **(B)** The cycle frequencies (Hz, and SD) in the pro-, meso-, or metathoracic ganglia under the different experimental conditions. Gray bars represent the rhythm induced by direct application of pilocarpine to the measured ganglion (when individually isolated or in the interconnected ganglia chain); white bars represent indirect pilocarpine activation. The different conditions are depicted in the pictogram at the bottom: shaded ganglia represent the directly pilocarpine-activated ganglion or ganglia under each condition. *P*-values are given for each thoracic ganglion above the bars (ANOVA or Kurskal–Wallis, see Materials and Methods for elaboration). Numbers in the bars represent the N values. Only the mesothoracic ganglion showed a difference in burst frequency, due to different application paradigms. **(C)** As in **(B)**, but for burst duration in seconds. None of the ganglia showed any difference among the different application methods. **(D)** As in **(B)**, but for duty cycle (burst duration relative to cycle period). Again, none of the ganglia showed any difference among the different experimental conditions.

To characterize the functional connectivity between each pair of CPGs we calculated the correlation coefficient from cross-covariance analysis (e.g., Borgmann et al., 2007), using MATLAB (MathWorks, USA Inc.). The analysis was based on the identification of spike (action potential) events only, and not bursts. The spikes were detected and identified by their amplitude and only the excitatory motor units (slow and fast trochanteral depressors) were taken into account, without separating between them (see recording of nerve 5A in **Figure 1A**).

Finally, we analyzed the same set of data with the less common, but powerful, cross-spectrum analysis in order to determine the phases between pairs of active CPGs, following a procedure developed by Miller and Sigvardt (1998; see also Sigvardt and Miller, 1998). By using cross-power spectral density function in MATLAB (MathWorks, USA Inc.), based on Fourier (frequency-domain) time-series analysis, we determined the common frequencies in the paired recordings and their related phases. Additionally, we calculated the coherence between the paired recordings to statistically evaluate the entrained frequency bands (see for details: Sigvardt and Miller, 1998). Subsequently, we filtered the products of these operations to 0.05 and 0.4 Hz, thus excluding most non-bursting activity from the results, and selected only frequency bands that showed a significant coherence.

**FIGURE 2:**
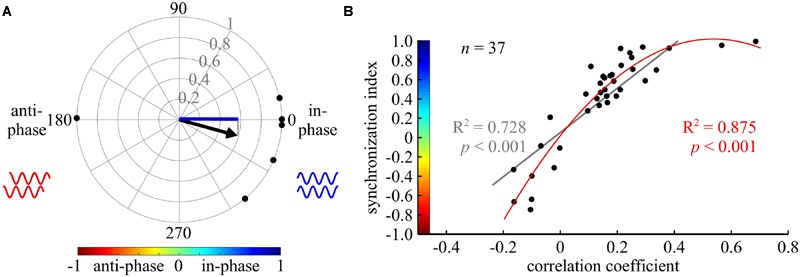
**Cross-spectrum analysis: a new tool for phase analysis in insect locomotion research. (A)** An example of the synchronization index calculation. The compass plot represents the bi-lateral phases of depressor MNs activity in six isolated prothoracic ganglia. The phase in each experiment is represented by a black point on the circle perimeter, calculated by the cross-spectrum analysis. The mean vector of all experiments is indicated by the black arrow. The synchronization index, symbolized by the blue line, is the projection of the mean vector on the 0–180 degrees axis. Thus, it represents the synchrony level of the six experiments together. In the following figures, synchronization index is indicated by the color code, representing both its magnitude and direction, as shown in **(B)**. **(B)** A scatter plot illustrating the relationship between the results of a cross-covariance analysis (horizontal axis), and the synchronization index (vertical axis, with implemented color code). Both a linear and a second degree polynomial fit were calculated (gray and red lines and regression results, respectively).

For each pair of recordings we averaged the phases related to the filtered frequency bands using the circular statistic toolbox in MATLAB (Berens, 2009). The mean phases were then averaged again for all experiments. The Watson–Williams *F* test was used to test for differences in the phase vectors. Since no set of experiments showed a clear phase other than 0° (in-phase) or 180° (anti-phase), we used the synchronization index to determine the kind of coupling between CPGs, and its relative strength. The synchronization index was calculated by projecting the mean phase vector of all experiments onto the 0–180° axis (see **Figure 2A**). Thus, the synchronization index represents quantitatively both the significant coupling direction and its variation. First and second degree polynomial fitting were used in order to investigate the relation between the synchronization index and the cross-covariance correlation coefficient, and to further validate the use of the former parameter (**Figure 2B**).

## Results

Due to the vast modulatory proprioceptive inputs affecting the motor control of walking, deprivation from all sensory inputs is required in order to study the functional central interconnectivity of leg CPGs (for reviews: Büschges, 2005; Ayali et al., 2015a,b). Therefore, in the following experiments we used an *in vitro* isolated locust nervous system preparation, comprising the three thoracic ganglia. Motor patterns were activated by application of the muscarinic agonist pilocarpine (see next section). To determine the inter-CPG connectivity we used the following isolated preparations and activation methods: (1) individual thoracic ganglia activated with pilocarpine directly (individual ganglion – direct activation); (2) three interconnected thoracic ganglia, activated simultaneously with pilocarpine (whole chain – direct activation); and (3) three interconnected thoracic ganglia, in which one ganglion – either the pro-, meso-, or metathoarcic ganglion resting in a Vaseline-constructed chamber – was separately activated with pilocarpine, while the two other ganglia were not exposed to the pharmacological agent (whole chain – restricted activation).

### Pilocarpine Activates Leg CPGs

Pilocarpine has been previously shown to activate invertebrate leg CPGs (Chrachri and Clarac, 1987; Ryckebusch and Laurent, 1993, 1994; Ryckebusch et al., 1994; Büschges et al., 1995; Johnston and Levine, 2002; Fuchs et al., 2011, 2012; Rillich et al., 2013). **Figure 1A** illustrates a typical rhythmic burst pattern induced by 5^∗^10^-4^ M pilocarpine, as recorded from an isolated meta-thoracic ganglion. The simultaneous extracellular recording presents alternating activity of the trochanteral depressor (nerve 5A) and levator (nerve 3B and 4A) motor neurons (MNs) that participate, respectively, in the leg stance and swing phases during walking. Due to the robust and very consistent pattern of these antagonistic MN pools, in the following experiments we evaluated only the bursting activity of depressor MN pools in order to study the interplay between the coxa-trochanteral CPGs of different legs.

### Active Leg CPGs Can Recruit CPGs in Other Segments

Functional connections among CPGs can potentially mediate two types of interactions: (1) the activation of one oscillator by another; and (2) the temporal or phasic entrainment of one oscillator by another. We used the different isolated preparations in order to uncover CPG-CPG interactions.

Confirming earlier reports (Rillich et al., 2013), under all experimental conditions (individual ganglion – direct activation, whole chain – direct activation, whole chain – restricted activation), prior to any pilocarpine application we observed either no activity, or slow tonic firing of the slow depressor MN (*N* = 57 experiments). Following direct pilocarpine application, either to individual ganglia or to the whole chain, a typical bursting pattern was generated, which started within the 1st minute and persisted throughout the length of the experiment (40 or more minutes), without any noticeable perturbations (individual ganglia *N* = 20, whole ganglia *N* = 13). Similarly, in the whole-chain restricted activation experiments, the pharmacologically treated ganglion started bursting within 1 min post-application. Remarkably, in all restricted application experiments (*N* = 25 experiments), we also observed bursting activity of all CPGs in the non-drug-treated ganglia. This indirect activation began within 5 min post-application and persisted throughout the experiment. Interestingly, the capacity of one stimulated ganglion to induce activity in the other ganglia was equal among the pro-, meso-, and metathoracic ganglia.

Previous studies have shown that the leg CPGs do not have an internal fixed bursting rhythm, as their characteristics change with different concentrations of pilocarpine (e.g., Rillich et al., 2013). Therefore, we further sought to determine whether the different pilocarpine application procedures would result in different bursting patterns in any of the recorded ganglia. Hence, we compared the cycle frequency, burst duration, and duty cycle among the experimental conditions (**Figures 1B–D**). All comparisons resulted in no significant differences (**Figures 1B–D**), except for the cycle frequency of the mesothoracic ganglion (Kruskal–Wallis test: *H* = 21.53; *p* < 0.001, **Figure 1B**).

We were further interested in determining whether the restricted application procedures would result in similar bursting frequencies in the ganglion to which pilocarpine was applied and in the drug-free ganglia. Throughout the “whole-chain – restricted activation” experimental sets, all cycle frequencies observed in the untreated ganglia resembled those of the pilocarpine-activated ganglion: the experimental averages of the frequencies of the untreated ganglia were 99–116% of the averaged frequencies of the pilocarpine-activated ganglion (data not shown).

Our results demonstrate that the active leg CPGs in each of the thoracic ganglia are equally capable of recruiting all other CPGs in the other, indirectly stimulated, ganglia. The resultant bursting activity in the untreated ganglia, in almost all cases, did not differ from that induced by direct pilocarpine application, and was presumably generated or enforced by the pilocarpine-stimulated ganglion.

### Cross-Spectrum Analysis for Oscillator Coupling Evaluation

The capacity of active leg CPGs to initiate and maintain activity in other ganglia CPGs indicated the existence of functional connections among them. We therefore sought to further determine whether phasic information is also transferred among these CPGs to shape their orchestration.

To do so, we used cross-spectrum analysis to quantify the phase between each pair of CPGs, and a coherence measurement to evaluate the degree of entrainment (see details in the Materials and Methods section, and Miller and Sigvardt, 1998 for further information). Only significantly entrained frequency bands (as indicated by the coherence values) were used to calculate mean vectors for each experiment (Supplementary Tables 1–3). Therefore, in a few cases in which no significant coherence was found, the number of experiments (N) was larger than the number of CPG pairs tested for coupling strength (n). Finally, to describe both the direction and variance of the phase in one value, a synchronization index was defined as the projection of the mean vector on the in-phase-anti-phase axis, yielding values from 1 (perfect in-phase) to -1 (perfect anti-phase). **Figure 2A** illustrates the calculation of the synchronization index.

**FIGURE 3:**
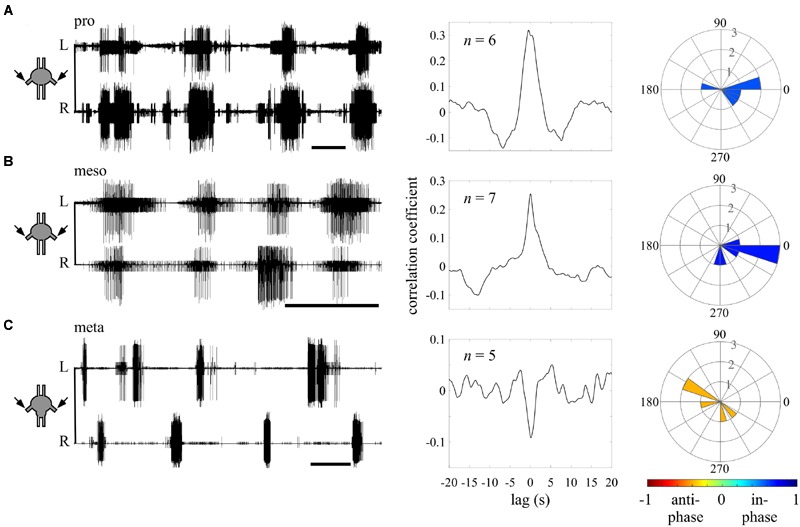
**Different endogenous bilateral coupling among the different thoracic ganglia revealed through direct pilocarpine applications to individual ganglia. (A)** Left: simultaneous extracellular recordings of burst activity in the left (L) and right (R) depressor MNs of an isolated prothoracic ganglion following pilocarpine application (scale bars: 5 s). Middle: cross-covariance result illustrating a dominant positive peak at a zero lag. The *n*-values stand for the number of evaluated paired recordings after the coherence calculation filtering. Right: A circular histogram illustrating the bilateral phase calculated for each isolated ganglion. The phases of different experiments are binned in 18 degrees-wide bars. The color filling of the bars indicates the synchronization index calculated for the bilateral coupling of the entire experimental set (color scale at the bottom; see also **Figure 2**). **(B)** As in **(A)** but for the isolated mesothoracic ganglion. **(C)** As in **(A)** but for the isolated metathoracic ganglion. Note that the correlation coefficient and synchronization index consistently indicate an in-phase coupling between the pro- and mesothoracic hemiganglia and an anti-phasic coupling for the metathoracic hemiganglia.

This type of analysis has not previously been used in studies of walking motor patterns. Therefore, we compared the calculated synchronization index to the value of the frequently used cross-covariance at zero lag. As illustrated in **Figure 2B**, high first and second degree polynomial fittings were found, validating the use of the synchronization index in the study of walking (nine different CPG pairs were evaluated under five different conditions). For specific examples, compare the cross-covariance results and circular histograms in **Figure 3**. Use of the cross-spectrum analysis confers several benefits in comparison to other methods, as discussed by Sigvardt and Miller (1998), and briefly described in the “Discussion.” We therefore used this method throughout to characterize the phase relations between different CPGs.

### Intra-Segmental Coupling Is Segment-Specific

We first sought to evaluate the bilateral phase relations within each thoracic ganglion, when separated from the others. Therefore, we calculated the bursting phase between each pair of segmental oscillators in the “individual ganglion – direct activation” experiments (**Figure 3**). Both the pro- (*N* = 6, *n* = 6) and mesothoracic ganglion (*N* = 7, *n* = 7) left and right CPGs fired in-phase, with mean synchronization indexes of 0.589 and 0.708, respectively. In contrast, the metathoracic ganglion bilateral CPGs were active in alternation (*N* = 6, *n* = 5), with a mean synchronization index of -0.397.

Overall, the different thoracic ganglia demonstrate differential patterns of bilateral functional connectivity. Since these experiments were conducted on separated ganglia, we can refer to the obtained patterns as the inherent coupling of each ganglion, uninfluenced by functional connections to CPGs of other segments.

### Differential Inter- and Intra-Segmental Coupling in the Thoracic Ganglia Chain

**FIGURE 4:**
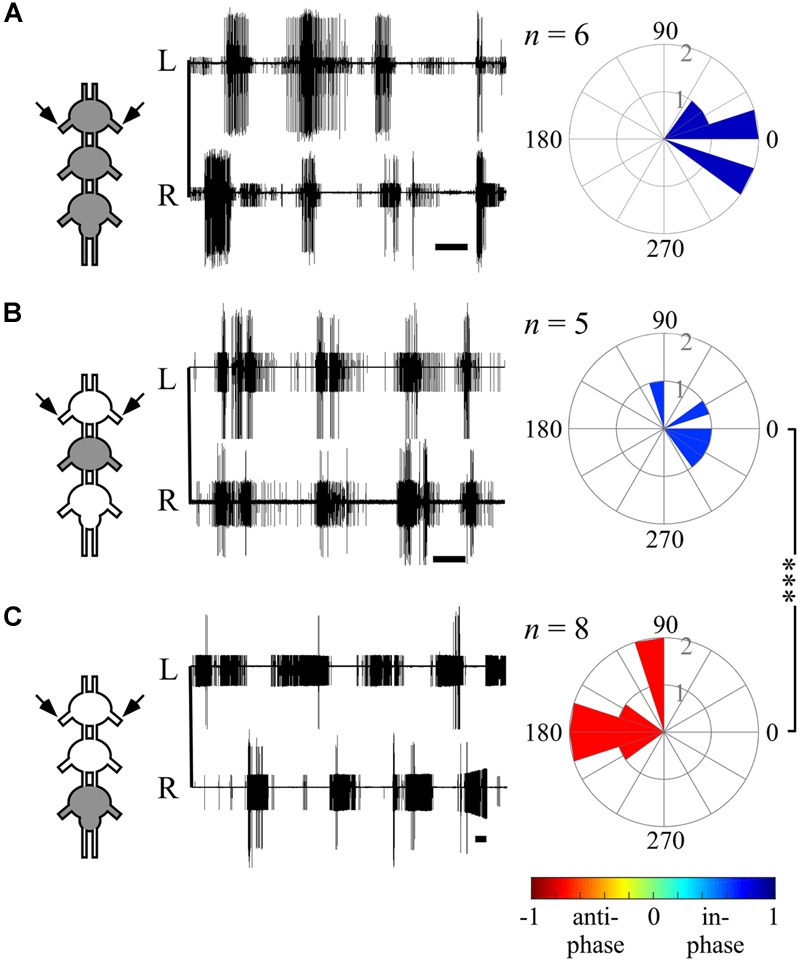
**Different bilateral coupling in the prothoracic ganglion induced by different Pilocarpine application paradigms. (A)** Left: simultaneous extracellular recordings of burst activity in left (L) and right (R) depressor MNs of the prothoracic ganglion in interconnected thoracic ganglia chain preparations following direct pilocarpine application to all interconnected ganglia (see pictogram on the left, Scale bars: 3 s). Right: circular histograms illustrating the phase between the prothoracic hemiganglia. The synchronization indexes are indicated by the filling color. **(B)** As in **(A)** but for restricted pilocarpine application to the mesothoracic ganglion only. **(C)** As in **(A)** but for restricted pilocarpine application to the metathoracic ganglion. Note that the prothoracic ganglion bilateral coupling pattern has changed as a function of the pilocarpine application method: from in-phase, synchronized bilateral activity for the direct pilocarpine application to all thoracic ganglia **(A)**, and the restricted application to the mesothoracic ganglion **(B)**, into anti-phasic activity under restricted application to the metathoracic ganglion **(C)**. The *n*-values represent the number of evaluated paired recordings. Watson Williams multiple sample test: ^∗∗∗^*p* < 0.001.

**FIGURE 5:**
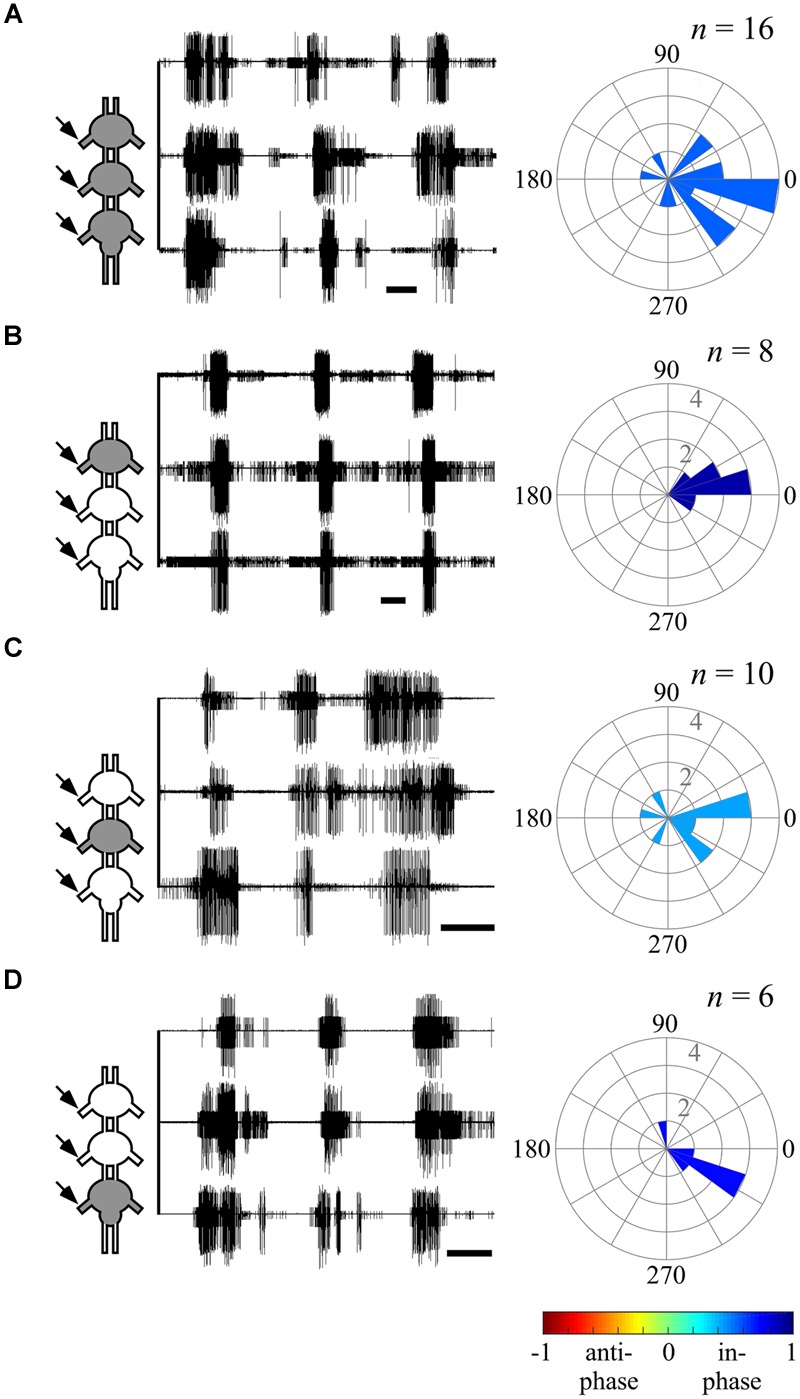
**Ipsilateral CPGs are strictly synchronized, independent of activation methods. (A)** Simultaneous extracellular recordings of the ipsilateral pro-, meso-, and metathoracic left hemiganglia depressor MNs after direct pilocarpine application to all thoracic ganglia (Scale bars: 3 s). The circular histogram illustrates the phase between the non-adjacent ipsilateral pro- and metathoracic ganglion. Filling color indicates the synchronization index. The number of evaluated paired recordings are noted above. **(B)** As in **(A)**, but for restricted pilocarpine application to the prothoracic ganglion. **(C)** As in **(A)**, but for restricted pilocarpine application to the mesothoracic ganglion. **(D)** As in **(A)**, but for restricted pilocarpine application to the metathoracic ganglion. Note that the synchronization indexes indicate in-phase coupling between the pro- and metathoracic ipsilateral hemiganglia irrespective of the pilocarpine application procedure.

In order to determine whether temporal or phasic information passes among the inter-segmental CPGs, we first applied the direct pilocarpine procedure to the whole interconnected thoracic ganglia chain. In accordance with previous findings in the stick insect (Büschges et al., 1995), under this condition we found synchronized activity of all CPGs in all three thoracic ganglia (see, for example, the bilateral coupling in the prothoracic ganglion in **Figure 4A** and ipsilateral inter-segmental coupling in **Figure 5A**). Interestingly, the bilateral coupling of the metathoracic ganglion, which was found to be inherently anti-phasic, was switched to in-phasic by the influence of the other ganglia activated CPGs. All synchronization indexes ranged from 0.362 to 0.877.

**FIGURE 6:**
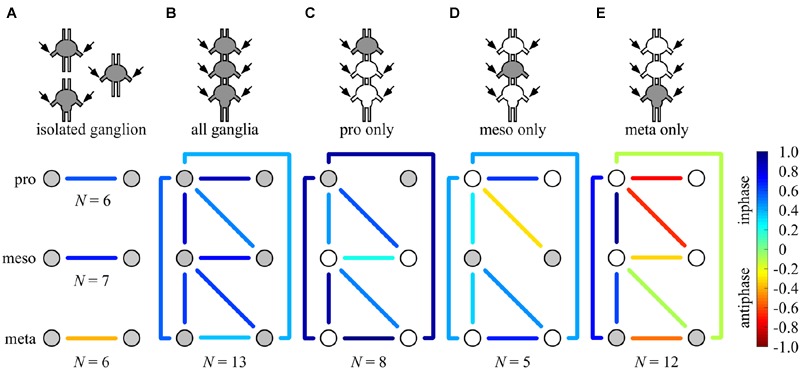
**Summary of all possible phase-relationships among the six thoracic CPGs under all experimental conditions.** The schemes illustrate the six recorded CPGs as circles (upper, middle, and bottom circles represents the pro-, meso-, and metathoracic ganglia, respectively) and their coupling by the color-coded lines (left–right symmetry is assumed), under the different experimental conditions: **(A)** individual ganglion – direct activation, **(B)** whole chain – direct activation, **(C)** whole chain – restricted activation of the prothoracic ganglion, **(D)** whole chain – restricted activation of the mesothoracic ganglion, **(E)** whole chain – restricted activation of the metothoracic ganglion. Overall, each ganglion is able to impose its own inherent coupling pattern (as shown in **A**) onto the others due to a dominance of ipsilateral synchrony. Number of preparations is indicated for each condition.

The ability of one ganglion to induce activity in other ganglia CPGs allowed us to investigate the functional connectivity of each thoracic ganglion to the others. Hence, we compared the phase relations between the different oscillators’ activity as induced by restricted pilocarpine application to each of the thoracic ganglia. When pilocarpine was applied to the prothoracic ganglion alone (*N* = 8), all monitored depressor MN pools thereupon became active in-phase, with synchronization indexes ranging from 0.211 to 0.995 in all three ganglia (Supplementary Tables 1–3, and see, for example, ipsilateral inter-segmental coupling in **Figure 5B** and the scheme in **Figure 6C**). Similarly, restricted application of pilocarpine to the mesothoracic ganglion resulted in an in-phase activity of almost all CPGs (see, for example, the bilateral coupling in the prothoracic ganglion in **Figure 4B**, ipsilateral inter-segmental coupling in **Figure 5C** and the scheme in **Figure 6D**; *N* = 5). Thus, in accord with the direct pilocarpine application to the whole chain, both pro- and mesothoracic active CPGs were able to induce bilateral synchronization in the pharmacologically untreated metathoracic ganglion (**Figures 6C,D**).

Of special interest are the results of the restricted pilocarpine application to the metathoracic ganglion, due to its unique anti-phase inherent coupling. We found that when the leg CPGs of the metathoracic ganglion were restrictively pharmacologically activated, the ipsilateral CPGs, both in the meso- and prothoracic ganglia, maintained in-phase activity (**Figures 5D** and **6E**). However, all contralateral CPG couples were drawn toward anti-phasic activity (**Figure 6E** and see, for example, the bilateral coupling in the prothoracic ganglion in **Figure 4C**). This finding was specifically intriguing with respect to the intrasegmental coupling of the pro- and mesothoracic ganglia, in which the mean synchronization indexes were -0.748 and -0.33, respectively. The anti-phasic bilateral activity of the restrictively pharmacologically activated metathoracic ganglion was confirmed as remaining similar to its inherent coupling pattern, as found in the “direct activation – individual ganglion” experiments (synchronization index = -0.637, *n* = 5, **Figure 6E**; Supplementary Table 1). **Figure 6** and Supplementary Tables 1–3 summarize all possible CPG-CPG connections of the different experimental sets (three bi-lateral intra-segmental, three ipsi-lateral inter-segmental, three contralateral inter-segmental).

Overall, we found that the different pilocarpine application procedures resulted in differential CPGs coupling schemes: while ipsilateral CPGs were active in-phase throughout all experimental sets, bilateral connections demonstrated flexibility, adopting the inherent coupling pattern of the restrictedly stimulated ganglion, and ranged between high in-phasic to high anti-phasic activity.

### Inter-Segmental Information Transfer Is Predominantly Ipsilateral

One possible explanation for the ability of each ganglion to impose its inherent bilateral coupling onto the other ganglia is that the two sides of the thoracic ganglia chain comprise two ipsilateral functional units, which dominate the bilateral couplings. Alternatively, it is also conceivable that contralateral ascending or descending interneurons provide an essential input for the activation of CPGs in other segments.

**FIGURE 7:**
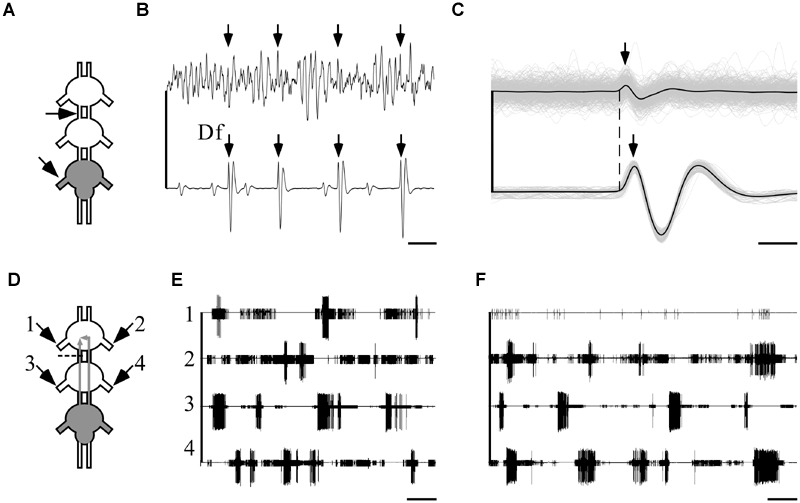
**Ipsilateral connections are sufficient and necessary for the maintenance of indirectly pilocarpine-induced pattern.** A pictogram illustrating the recording sites **(A)** and an example of a recording **(B)** from the pro-mesothoracic connective (top) and the ipsilateral metathoracic depressor nerve. Arrows indicate the fast depressor action potentials which are accompanied by a spike in the connective recording (scale bar: 10 ms). **(C)** An overlay of all correlated fast-depressor and pro-mesothoracic connective action potentials in an 8-min recording. The individual overlaid recordings are shown in gray, while the black lines represent their mean. The dashed line denotes the onsets of the fast depressor action potentials. Note that the activity in the connective is correlated to the occurrence of fast-depressor spikes (scale bar: 1 ms). **(D)** A pictogram illustrating the recording sites and the cut of the left connective between the pro- and mesothoracic ganglia in an interconnected ganglia chain experiment with restricted pilocarpine application to the metathoracic ganglion. The cut was designed to interfere with the ipsilateral connection. **(E)** Simultaneous extracellular recordings showing burst activity of depressor MN in both sides of the pro- (left: 1, right: 2) and the mesothoracic ganglia (left: 3, right: 4) following pilocarpine application as described in **(D)** (scale bar: 5 s). **(F)** As in **(E)**, but immediately after cutting the left connective as described in **(D)**. Note that after cutting the connective the bursting activity in the prothoracic hemiganglion ipsilateral to the cut was abolished, whereas the patterns of all other depressor MNs were unaffected, including the prothoracic hemiganglion contralateral to the cut (scale bar: 5 s).

In order to investigate these two options, we first monitored the ipsilateral-intersegmental activity in the pro-mesothoracic connective while pilocarpine was restrictively applied to the metathoracic ganglion (**Figures 7A,B**). Overlaying the recordings by fixing the onsets of the fast depressor action potentials revealed a distinct interneuronal activity in the connective that occurs simultaneously with the ipsilateral fast depressor activity (1080 action potentials, **Figure 7C**).

Next, in order to discriminate between the ipsi- and contralateral inter-segmental impacts, we severed one connective between the pro- and mesothoracic ganglia in five experiments in which pilocarpine application was restricted, as above, to the metathoracic ganglion only (**Figure 7D**). Before the cut, as reported above, all recorded depressor MNs showed rhythmic bursting activity (**Figure 7E**). Following the cut, the prothoracic hemiganglion ipsilateral to the cut stopped bursting immediately, in spite of the intact contralateral connections. All other recorded MN pools maintained their bursting rhythm, including the mesothoracic hemiganglion caudal to the cut and the prothoracic hemiganglion contralateral to the cut (**Figure 7F**).

These results demonstrate that the capacity of the metathoracic ganglion, and presumably all thoracic ganglia, to induce and maintain activity in the other ganglia CPGs is manifested via the ipsilateral connections alone and dependent on them. Furthermore, the findings demonstrate that each half of the thoracic ganglia chain constitutes a single functional unit that dominates the intra-segmental connections.

## Discussion

The neuronal organization behind insect walking comprises both sensory and central mechanisms of control (for reviews: Büschges, 2012; Ayali et al., 2015a,b). Previous work has tended to emphasize a peripheral-sensory point of view (e.g., Schmitz et al., 2001; Cruse, 2002). Our understanding of the CPGs organization, or the “central rules” of networks that are used for locomotion, is far from complete. Based on a series of complementary *in vitro* experiments and new and powerful analytical tools, here we deconstructed the network into its basic central elements and revealed that the functional connectivity among leg CPGs in the locust is capable of both co-activating and phasically entraining the network’s rhythmic output (**Figure 6**). One general common motif can be clearly seen throughout the various data presented: among the three segmental couples of oscillators, ipsilateral connections force strict synchronization, whereas bilateral connections show high modularity. Thus, the emerging coupling scheme offers both the rigid scaffold necessary for stereotyped movement and the central fundamentals for behavioral flexibility.

### The Use of Pilocarpine to Induce Motor Activity

Pilocarpine is widely used in the study of insect walking (e.g., Büschges et al., 1995; Borgmann et al., 2009; Fuchs et al., 2011), but nonetheless presents some problematic aspects. The full extent of pilocarpine influence on the insect nervous system is not clear, and when bath-applied to a ganglion it might activate different motor systems in parallel. Moreover, in rodents pilocarpine is used to model epilepsy (Cavalheiro, 1995), and it is possible that such epileptic-like seizures occur in insects too. From the perspective of insect walking, as Ludwar et al. (2005) rightly claimed, bath application of pilocarpine results in the co-activation of all leg CPGs directly and simultaneously, and thus might mask coupling patterns among them. Additionally, a common concern regarding any neuromodulation of *in vitro* preparations is the extent to which the produced activity is related to the natural behavior.

These problematic issues did not escape our attention. We used a pilocarpine concentration that is below the threshold necessary to activate flight CPGs (Rillich et al., 2013). Moreover, we have successfully shown for the first time that activating a single thoracic ganglion’s CPGs by pilocarpine is sufficient for the activation of the other, drug-free, leg CPGs (see previous unsuccessful attempt in stick insect, Ludwar et al., 2005; and a similar procedure used on abdominal ganglia for moth crawling, Johnston et al., 1999). The use of restricted pilocarpine application largely excluded some possible artifacts in the untreated ganglia, and thus gave us access to the rhythmic motor patterns of the CPGs when not directly treated pharmacologically. The comparison of motor outputs revealed almost no difference between direct pilocarpine application to a ganglion, and its indirect activation by restricted application to another ganglion (**Figures 1B–D**). This finding suggests that the pilocarpine-induced activity does not result in a non-physiological rhythm.

In addition to walking, the locust legs are used for jumping, swimming, kicking, searching, grooming, and righting (Rowell, 1964; Heitler and Burrows, 1977; Pflüger and Burrows, 1978a,b; Zill, 1985; Faisal and Matheson, 2001). Most of these behaviors are characterized by short duration, non-rhythmic activity, or by the involvement of only a limited number of legs. These features do not correspond with the type of activity we recorded. Swimming shares some characteristics with the data presented here. However, it shows high variability in the leg coordination pattern and in the number of active legs, and in general is not very common. While we cannot fully exclude the presented data from being related to any of the mentioned behaviors, we assume that the default central configurations of the legs as reported here is the product of hardwired neuronal connections that constitute the infrastructure for any leg-coordinated activity in the locust. As walking is the most prominent joint activity of the six legs, we further discuss our results from the locomotion perspective, in relation to previous studies that attributed pilocarpine-induced activity to walking (Borgmann et al., 2009; Fuchs et al., 2011, 2012; Rillich et al., 2013; David et al., 2016).

### Cross-Spectrum Analysis

Previous studies in insect walking have used mainly cross-covariance or cross-correlation and burst analysis to measure coupling between CPGs (e.g., Ryckebusch and Laurent, 1994; Borgmann et al., 2009). The two former methods provide good indication of the timing of the two signals, but fail to indicate their phase. Phase calculations based on burst analysis are often hampered by the unclear identification of bursts (Sigvardt and Miller, 1998). The resultant filtering could greatly affect the data-set. In the current study, we used the alternative cross-spectrum analysis method, originally designed for the lamprey swimming model (Miller and Sigvardt, 1998). This analysis overcomes both problems by taking the entire data-set into account and yielding accurate, Fourier-transform-based phase values for the different frequencies. Additionally, using coherence as an entrainment measurement, we implemented a statistical tool for filtering out irrelevant frequency bands.

Since the analysis of the data yielded no clear phases other than in-phase and anti-phase, we were able to use a synchronization index based on the cross-spectrum analysis. The benefit of this measurement lies in the quantification and representation of both the direction and the variance of the coupling in a single parameter. The high correlation between the synchronization index based on the cross-spectrum and cross-covariance analysis (**Figure 2B**) provides an empirical validation for the use of this novel tool. We thus recommend the use of cross-spectrum analysis for evaluating CPG coupling, and specifically in studies of walking.

### The Flexible Elements of the Motor Circuits

Three neuro-anatomical units – the three thoracic ganglia – encompass the three pairs of coxal-trochanteral CPGs. By deconstructing the system into these elementary parts and applying pilocarpine to each, we were able to reveal the inherent bilateral coupling pattern of the segments unaffected by inter-segmental connections. Intriguingly, we revealed that the three units are not identical: when separated from the other ganglia, the pro- and mesothoracic ganglia depressors burst in left-right synchrony, whereas those of the metathoracic ganglion bursts in alternation (**Figure 6A**).

Most legged locomotion involves a high degree of leg specialization, and hence longitudinal heterogeneity is likely. In the locust, beyond their role in walking, the hind legs are used for jumping and accordingly differ structurally from the two rostral pairs of legs. Furthermore, anatomically, the metathoracic ganglion is fused together with three abdominal ganglia, which could affect the thoracic central patterns, as shown in cricket song production (Schöneich and Hedwig, 2011). Some studies in insects have already indicated that the metathoracic motor activity differs functionally from that of the pro- and mesothoracic ganglia: Bässler et al. (1985) showed that the inherent direction of stepping of the stick insect hind legs is backward, whereas the front legs naturally walk forward, and a recent study on fruit flies identified metathoracic ganglion neurons that induce backward-walking (Bidaye et al., 2014). Fuchs et al. (2011), who studied the cockroach, found that the descending oscillator entrainment is stronger than the ascending one, suggesting another asymmetry in the system (see also David et al., 2016). However, none of the above-mentioned studies supply a satisfactory explanation for the unique alternating pattern of the locust metathoracic ganglion. Our results further show that the variable bilateral connections provide the substrate for two different network configurations of the ganglia chain motor output, as discussed below.

We found that each ganglion can impose its own inherent coupling pattern onto the others: pharmacological activation of the pro- or mesothoracic ganglia resulted in the synchronization of all CPGs within the network, whereas following restricted application to the metathoracic ganglion, all segmental bilateral CPGs were active in left-right anti-phase (compare **Figures 6C–E**). This finding reveals that the bilateral coupling of each segment is flexible, and can be overwritten by the intersegmental interneurons driven by other segments’ CPGs.

The activation of all ganglia simultaneously resulted in an in-phase activity of all CPGs. This outcome seems to be the result of an internal competition between descending and ascending inputs from the different ganglia, in which the sum of inputs from the bilateral synchronized pro- and mesohoracic ganglia overrides the anti-phasic inherent pattern of the metathoracic ganglion.

Overall, our findings indicate that while each ganglion possesses an inherent activity pattern, the segmental bilateral couplings are highly modular. These flexible network elements provide the medium for the pluripotentiality of the network motor outcome, even in the absence of sensory input and descending control. Thus, a differential activation of ganglia with opposing inherent coupling patterns could determine the overall bilateral coupling.

### The Rigid Elements of the Motor Circuits

The ability of each ganglion to impose its own coupling pattern onto the other ganglia suggests robust and consistent inter-segmental functional connections, based on ascending and descending interneurons (e.g., **Figures 7A–C**). Consequently, we were not surprised to find that throughout all our experimental sets, ipsilateral CPG activity was always synchronized (**Figure 6**). Accordingly, by cutting one connective we showed that ipsilateral coupling is both sufficient and necessary for maintaining pattern-activity in leg CPGs, whereas bilateral connections alone are not sufficient to maintain activity in pharmacologically untreated CPGs (**Figures 7D–F**). This finding resembles that demonstrating the independency of each side of the tadpole spinal cord in producing a coordinated swimming rhythm (e.g., Li et al., 2010, but see Moult et al., 2013).

Previous studies in the stick insect and cockroach have shown that one stepping leg is capable of entraining the CPGs of neighboring segments (Borgmann et al., 2007; Fuchs et al., 2011). We have shown here that each of the functional unit’s CPGs can equally activate and entrain the other members of the lateral rigid unit: both the descending inputs of interneurons from the prothoracic ganglion and the ascending inputs of interneurons from the metathoracic ganglion were sufficiently strong to activate and entrain rhythmic pattern in adjacent and non-adjacent thoracic ganglia. Furthermore, while the middle legs had been previously described as dominated by the other segments (Bässler et al., 1985; Graham and Epstein, 1985; Borgmann et al., 2009), in the present study the mesothoracic CPGs demonstrated the same ability to induce and maintain activity within the lateral units.

Taken together, these findings indicate that each of the two sides of the thoracic ganglia chain comprises an inherently rigid functional unit, which can both recruit its members and synchronize their bursting activity. In natural walking, inter-leg temporal entrainment is essential for coordinated movements and for instantaneous concerted changes in walking speed (Wendler, 1964; Graham, 1972; Graham and Cruse, 1981; Gabriel and Büschges, 2007; Wosnitza et al., 2013). Correspondingly, we have shown that the rigid lateral units can disperse similar bursting parameters in all CPGs (**Figures 1B–D**), and thus possibly confer the inter-leg uniformity needed for the orchestration of different walking maneuvers. Following Büschges et al. (1995), the physiologically unintuitive synchrony that the lateral units sustain can be explained by energy calculation, as it is energetically cheaper to entrain oscillators in in-phase rather than in anti-phase (*Magnet-effect*, Holst, 1936).

### Rigid and Flexible Elements Synthesis in the Control of Walking

We have demonstrated here that the network of CPGs that control locust walking is composed of both endogenously rigid and flexible connections. We suggest that the rigid ipsilateral parts entrain the leg oscillators at each side to work together in orchestration similarly to a timing belt, which orchestrates the opening of the engine’s valves. The bilateral flexible parts, in contrast, show variability, and allow some independency to each lateral unit. Thus they introduce modularity into the overall coordination. A differential control of each group of lateral legs can serve as a basic feature of walking flexibility, and possibly enables the different movements of each side’s limbs during turning.

Our results offer several candidate leverage points that could be targets for the integration of locomotive commands. Previously, higher motor centers, such as the central complex and the subesophageal ganglion (SEG), were shown to control walking initiation, maintenance, turning, and speed (Kien and Williams, 1983; Bender et al., 2010; Libersat and Gal, 2013; Martin et al., 2015), but their interaction with the leg motor circuits remained unclear. Based on our current results, motor regulators can potentially reinforce the different ganglia in changing the bilateral tendency of the network, or differentially alter the oscillating parameters of each side of the ganglia chain independently in order to induce lateral asymmetry. In this context we are currently exploring the integration of the SEG into the CPG network, and our preliminary results suggest that it is instrumental in modulating the bilateral coupling of the thoracic ganglia chain.

### CPG Coupling and Sensory Information Integration

A strong central coupling between CPGs is found in both vertebrates and invertebrates, and in some cases it is capable of generating an activation pattern that already shares the main characteristics of the behavior that the networks control (fictive swimming: Grillner et al., 1995; Miller and Sigvardt, 2000; Cangiano, 2005; Moult et al., 2013; fictive crawling: Johnston and Levine, 1996; fictive flight: Wilson, 1961; Stevenson and Kutsch, 1987; fictive walking in rodents: Kiehn, 2011). Most insects walk in a tripod gait, in which each two extreme legs of one side and the middle contralateral leg move in-phase, and in a complete anti-phase with the other three legs. Yet, other gaits are also common in slow speed (Grabowska et al., 2012). The ability of the central connections among leg CPGs to dictate functional walking gaits seems to differ between species. Central couplings appear to be weak in the slowly walking stick insects (Büschges et al., 1995; Ludwar et al., 2005), whereas in the cockroach they are sufficient to generate a fictive walking gait (Fuchs et al., 2011; David et al., 2016). In the present study we have revealed different interconnectivity patterns among the leg CPGs in the locust. However, it is important to note that none of the activity patterns we observed represent a functional (fictive) walking gait.

The CPG infrastructure provides a substrate for sensory modulation to ultimately form a functional walking gait. One common feature to all hexapod leg coordination is the anti-phasic activity of one leg in regard to its ipsilateral legs (Ayali et al., 2015a,b). It is the role of sensory inputs, for example, to unsynchronize the ipsilateral synchronized trinity. As previously suggested, a likely candidate for such modification is the local load proprioceptive feedback loop (Borgmann et al., 2009; see also for proprioception: Bässler et al., 1985; Zill, 1985; Fuchs et al., 2012; Couzin-Fuchs et al., 2015).

## Conclusion

Insect walking is a highly complex operation that involves the coherent co-activity of 18 different CPGs, one for each joint of each leg (Büschges et al., 1995). The mechanism controlling the legs must supervise the orchestration of these joints, monitor interruptions in real time, and adapt immediately. Our results show that the leg CPG network features both rigid and modular elements that presumably allow both stereotyped walking movements and the flexibility to adapt to environmental and decisional requirements.

## Author Contributions

DK, AA, H-JP, and JR designed the study and wrote the manuscript. DK and JR conducted the experiments and analyzed the data.

## Conflict of Interest Statement

The authors declare that the research was conducted in the absence of any commercial or financial relationships that could be construed as a potential conflict of interest.
