# Supplementary material for: Rigidity and Flexibility: The Central Basis of Inter-Leg Coordination in the Locust
Source: Front Neural Circuits. 2017 Jan 11;10:112. doi: 10.3389/fncir.2016.00112 (PMC5225121; doi:10.3389/fncir.2016.00112)
Supplement: Supplementary file 1 [file Data_Sheet_1.pdf]

Supplementary Material

Rigidity and Flexibility: The Central Basis of Inter-Leg Coordination in the Locust

Daniel Knebel, Amir Ayali*, Hans-Joachim Pflüger, and Jan Rillich

*** Correspondence:** Amir Ayali ayali@post.tau.ac.il;

1.1 Table 1

Synchronization index (sync. index), mean phase, mean vector length (r), and number of evaluated paired recordings (*n*) for intra-segmental (left-right, l-r) CPG-CPG relationships in pro-, meso-, and metathoracic ganglia. Bi-lateral coupling was analyzed in single isolated ganglia and in interconnected thoracic ganglia chain preparations after direct application of pilocarpine or restricted pilocarpine application to only one of the thoracic ganglia. The synchronization indexes are depicted in Fig. 6.

CPG-CPG relationship	Parameter	Individual ganglion	Whole chain, pilocarpine applied to			
			All ganglia	Pro only	Meso only	Meta only
Pro l - r	*sync. index*	0.589	0.877	-	0.65	-0.748
	phase	0.957	0.015	-	0.998	0.446
	r	0.612	0.881	-	0.65	0.793
	n	6	6	-	5	8
Meso l - r	*sync. index*	0.708	0.747	0.211	-	-0.33
	phase	0.922	0.023	0.1	-	0.584
	r	0.803	0.755	0.262	-	0.383
	n	7	6	8	-	8
Meta l - r	*sync. index*	-0.397	0.362	0.995	0.699	-0.637
	phase	0.57	0.921	0.998	0.995	0.524
	r	0.438	0.412	0.995	0.7	0.645
	n	5	7	7	5	5

1.2 Table 2

Synchronization index (sync. index), mean phase, mean vector length (r), and number of evaluated paired recordings (*n*) for ipsilateral CPG-CPG relationships between pro-meso-, pro-meta-, and meso-metathoracic hemiganglia. Inter-segmental coupling was analyzed in thoracic ganglia chain

preparation after pilocarpine application to all ganglia or restricted application to only one of the thoracic ganglia. The synchronization indexes are depicted in Fig. 6.

CPG-CPG relationship	Parameter	Whole chain, pilocarpine applied to			
		**All ganglia**	**Pro only**	**Meso only**	**Meta only**
**Pro-meso**	*sync. index*	0.829	0.451	0.277	0.957
	phase	0.987	0.957	0.064	0.003
	r	0.832	0.468	0.301	0.957
	n	18	8	3	10
**Pro-meta**	*sync. index*	0.562	0.921	0.428	0.738
	phase	0.971	0.029	0.952	0.966
	r	0.571	0.937	0.448	0.756
	n	16	8	10	6
**Meso-meta**	*sync. index*	0.639	0.927	0.335	0.617
	phase	0.963	0.024	0.817	0.9276
	r	0.657	0.938	0.82	0.687
	n	17	17	2	18

1.3 Table 3

Synchronization index (sync. index), mean phase, mean vector length (r), and number of evaluated paired recordings (*n*) for contralateral (left-right, l-r) CPG-CPG relationships between pro-meso-, pro-meta-, and meso-metathoracic ganglia. Inter-segmental coupling was analyzed in thoracic ganglia chain preparation after direct pilocarpine application to all ganglia or restricted application to only one of the thoracic ganglia. The synchronization indexes are depicted in Fig. 6.

CPG-CPG relationship	Parameter	Whole chain, pilocarpine applied to			
		**All ganglia**	**Pro only**	**Meso only**	**Meta only**
**Pro-meso l - r**	*sync. index*	0.496	0.585	-0.311	-0.664
	phase	0.009	0.128	0.329	0.529
	r	0.497	0.844	0.654	0.675
	n	11	8	2	11
**Pro-meso l - r**	*sync. index*	0.404	0.938	0.428	-0.11
	phase	0.977	0.021	0.995	0.309
	r	0.408	0.947	0.428	0.303
	n	13	7	10	7
**Meso-meta l - r**	*sync. index*	0.632	0.497	0.467	-0.085
	phase	0.975	0.996	0.031	0.674
	r	0.639	0.497	0.476	0.185
	n	13	15	3	13
